# Fine-Tuned Expression of Evolutionarily Conserved Signaling Molecules in the *Ciona* Notochord

**DOI:** 10.3390/ijms252413631

**Published:** 2024-12-20

**Authors:** Lenny J. Negrón-Piñeiro, Yushi Wu, Ravij Mehta, Julie E. Maguire, Cindy Chou, Joyce Lee, Chitra L. Dahia, Anna Di Gregorio

**Affiliations:** 1 Department of Molecular Pathobiology, New York University College of Dentistry, 345 E 24th Street, New York, NY 10010, USA; 2 Orthopedic Soft Tissue Research Program, Hospital for Special Surgery, New York, NY 10021, USA; 3 Department of Cell and Developmental Biology, Weill Cornell Medicine, Graduate School of Medical Science, New York, NY 10065, USA

**Keywords:** ascidian, *Brachyury*, *Ciona*, *cis*-regulatory module, *Ctgf*, enhancer, notochord, *Rac1*, semaphorin, signaling molecule, *Tgf-β*

## Abstract

The notochord is an axial structure required for the development of all chordate embryos, from sea squirts to humans. Over the course of more than half a billion years of chordate evolution, in addition to its structural function, the notochord has acquired increasingly relevant patterning roles for its surrounding tissues. This process has involved the co-option of signaling pathways and the acquisition of novel molecular mechanisms responsible for the precise timing and modalities of their deployment. To reconstruct this evolutionary route, we surveyed the expression of signaling molecules in the notochord of the tunicate *Ciona*, an experimentally amenable and informative chordate. We found that several genes encoding for candidate components of diverse signaling pathways are expressed during notochord development, and in some instances, display distinctive regionalized and/or lineage-specific patterns. We identified and deconstructed notochord enhancers associated with *TGF-β* and *Ctgf*, two evolutionarily conserved signaling genes that are expressed dishomogeneously in the *Ciona* notochord, and shed light on the *cis*-regulatory origins of their peculiar expression patterns.

## 1. Introduction

The ascidian *Ciona* provides a rare blend of experimental advantages for studies of the notochord, the embryonic structure that supports and patterns the developing body of all chordates. The translucency of the *Ciona* embryo allows for the immediate visualization of the notochord and its precursors [1]. Furthermore, the rapid development, the ease of transgenesis, and the compact, fully annotated genome contribute to rendering the *Ciona* embryo uniquely suited for the identification of genes expressed during notochord morphogenesis and the analysis of their regulatory sequences [2,3]. Most importantly, the *Ciona* notochord expresses evolutionarily conserved transcription factors (TFs), including orthologs of the main drivers of notochord formation, Brachyury and Foxa2, among numerous others [4,5,6,7,8,9,10,11].

Even though the *Ciona* notochord is composed of only 40 post-mitotic cells, these cells derive from precursors of two distinct lineages; from anterior to posterior, the first 32 cells constitute the A-lineage (also known as the primary notochord), while the remaining 8 cells, which are all located at the posterior end of the tail, compose the B-lineage (also known as the secondary notochord) (e.g., [12]). The *Ciona robusta* genome (formerly *Ciona intestinalis* type A [13]) contains orthologs of vertebrate genes involved in the Hedgehog, Jak/Stat, NFκB, Notch, RTK/Ras/MAPK, TGF-β, and Wnt signaling pathways [6,14]. The expression of 109 of these genes has been analyzed in detail throughout development by whole-mount in situ hybridization (WMISH) [6]. Subsequently, the regulatory relationships among genes encoding for signaling molecules, their products, and those of genes encoding for TFs, have been analyzed by microinjecting in *Ciona* zygotes sequence-specific morpholino oligonucleotides [7]. Additional studies have focused on individual signaling pathways and have uncovered the pivotal role played in notochord induction by the translocation of β-catenin to the nuclei of mesendodermal precursors, which elicits the expression of the TF FoxD [15]. In turn, FoxD activates the expression of another TF, ZicL, the main regulator of the early expression of the TF *Ciona Brachyury* (*Ci-Bra*) in the primary notochord [15,16]; on the other hand, *Ci-Bra* expression in the secondary notochord depends upon the Notch/Su(H) signaling pathway [17,18]. Additional pathways involved in notochord induction are FGF/MAPK/MEK/ERK and Nodal [19,20]; ultimately, these pathways converge on the *Ci-Bra cis*-regulatory region, through TFs of the Ets [21,22] and Hes families [18], respectively. After notochord specification is completed, notochord precursors continue to divide until they form two rows of 20 cells each, which undergo medio-lateral intercalation to form the single rod of 40 definitive cells, a process that requires Ephrin signaling to be properly completed [23]; this crucial morphogenetic movement is controlled by the planar cell polarity (PCP) pathway [24], whose components are asymmetrically localized within notochord cells [25], and by FGF3 emanating from the nerve cord cells overlying the notochord [26]. Following intercalation, notochord cells undergo tubulogenesis [27,28], which leads to the formation of a fluid-filled lumen in the center of the notochord [28,29]; the subsequent lumen expansion is controlled by TGF-β through the Rho-associated protein kinase (ROCK) and actomyosin contractility cascade [29]. In addition to that, the expression of Rac1 and Rho GTPases and their involvement in cellular contractility had been reported in the *Ciona* notochord [30], although their precise windows of expression and the representation of additional components of the Rac1 signaling pathway remained unclear.

A characteristic unevenness in gene expression has been reported among the 40 notochord cells of *Ciona*, whereby some genes are expressed in a lineage-independent mosaic pattern [31], while others are either expressed in an anterior–posterior gradient, or at the tips of the notochord, or display hybridization signals of variable intensity between cells of the primary and secondary lineage [6,32,33]. Of note, some of these unevenly expressed genes encode for signaling molecules, including *Ciona transforming growth factor beta* (*TGF-β*; gene model: KH.C3.724), which is expressed in a posterior-to-anterior gradient [6,32], and *connective tissue growth factor* (*Ctgf*; gene model: KH.C9.172; also known as *cellular communication network factor 2*, abbreviated as *ccn2*), which is expressed in a mosaic pattern [32,34].

In vertebrates, the notochord gives rise to the cells of the *nucleus pulposus* (NP), which form the core of the intervertebral discs [35] (reviewed by [36]); the NP cells of the post-natal intervertebral discs continue to express Brachyury and other evolutionarily conserved TFs, signaling molecules, and structural proteins, some of which are also expressed in the *Ciona* notochord [10,37,38,39]. The functions of Rac1, TGF-β, and Ctgf have been analyzed in these structures in different vertebrates. Mouse embryos lacking *Rac1* in the epiblast display an abnormal development of the posterior notochordal plate, among other defects [40], while *TGF-β1* null mice have a high inflammatory response and high mortality in the neonatal stage [41,42]. TGF-β signaling is important for the patterning of intervertebral discs in mouse embryos, as the conditional deletion of its receptor *TGFbr2* impacts vertebral patterning [43]. The role of Ctgf in notochord formation has been studied in zebrafish, where embryos injected with morpholino oligonucleotides against Ctgf/Ccn2 display a distorted notochord and consequent failure in tail elongation, which leads to decreased survival [44]. In mice, the *Ccn2* null mutation causes skeletal defects by impairing cartilage development [45]. Surprisingly, the *NotoCre*-mediated conditional deletion of *Ccn2* in the mouse node showed only minor changes in the newly formed NP and intervertebral discs [46]; however, an accelerated degeneration of the intervertebral discs was reported at 17 months of age, indicating the importance of this gene for disc homeostasis [46].

In this study, we uncovered the expression of putative components of signaling pathways in the *Ciona* notochord, assessed their evolutionary conservation, highlighted lineage- and/or position-related differences in their expression levels across the notochord, and investigated whether these differences could be traced back to *cis*-regulatory sequences.

## 2. Results

### 2.1. Expression of Evolutionarily Conserved Signaling Molecules in the Ciona Robusta Notochord: Candidate Components of the Rac1 Signaling Pathway

In this study, we used whole-mount in situ hybridization (WMISH) on *Ciona robusta* embryos at developmental stages ranging from 110-cell to late tailbud [47] to elucidate the notochord expression of candidate genes that were identified through the analysis of transcriptomic studies or by orthology to vertebrate notochord genes. The WMISH results revealed the expression of 20 genes with ontologies related to signaling pathways in the developing *Ciona* notochord (Table S1); orthologous human genes were identified by integrating the annotations of *C. robusta* genes provided by the Ghost database (http://ghost.zool.kyoto-u.ac.jp/ accessed January–June 2024) [48] and Aniseed (https://www.aniseed.fr/ accessed January–June 2024) [49] database with the results of reciprocal BLAST and TBLASTN searches [50]. Gene ontologies (GOs) were assigned by cross-referencing individual GOSlim, GO (Orthology), GO (Blast), and GO (InterPro scan) entries provided by Aniseed with the information contained in Gene Cards [51] and with related publications. Table S1 also reports any additional previously published data available for the pre- and post-metamorphic expression of each of the 20 genes, and lists their presumed transcriptional activators, which were determined on the basis of the results of occupancy studies [52] and misexpression assays [53,54].

**Figure 1:**
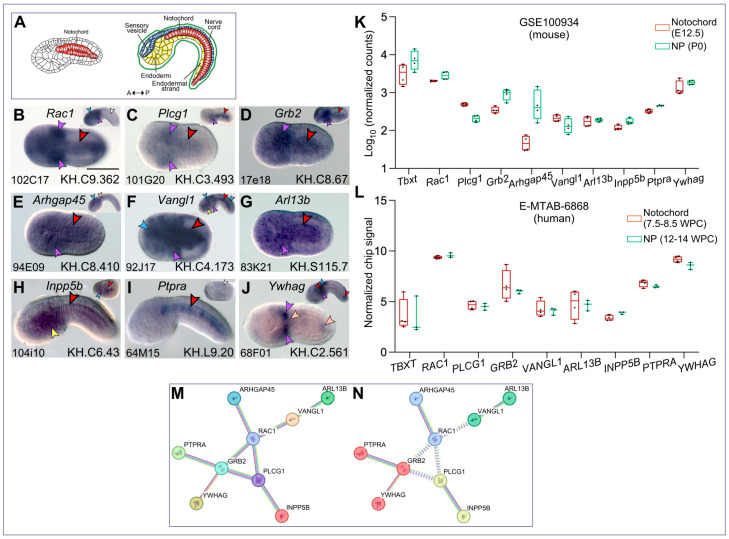
Reconstruction of the Rac1 interactome in the *Ciona* notochord. (**A**) Drawings of *Ciona robusta* embryos at the initial tailbud (**left**) and late tailbud (**right**). In the initial tailbud, only the notochord cells are colored (red). In the late tailbud, also the CNS (sensory vesicle and nerve cord), epidermis, and endoderm (trunk endoderm and endodermal strand) are colored (blue, green, and yellow, respectively), while tail muscles and mesenchyme, which fall onto different focal planes, are not depicted. Abbreviations: A, anterior; P, posterior. (**B**–**J**) Whole-mount *C. robusta* embryos at the initial tailbud stage (**B**–**G**,**J**; dorsal view) and mid-tailbud II (**H**,**I**; side view), hybridized in situ with digoxigenin-labeled antisense RNA probes. Gene names are reported on top of each panel; gene models on the bottom right corners; on the left corners, the ESTs used to synthesize each antisense probe are reported. Insets in (**B**–**F**,**J**) display embryos at tailbud stages subsequent to those in the main panels; inset in (**H**) shows an embryo at the late gastrula stage; inset in (**I**) shows an embryo at the late neurula stage. In all panels, anterior is to the left. Arrowheads color code: red, notochord; pink, faint notochord signal; white, unstained notochord; blue, CNS; violet, mesenchyme; yellow, endoderm. Scale bar: 50 μm. (**K**) Graph of the normalized read counts from the notochord of E12.5 mouse embryos (E12.5) and from NP cells at post-natal day zero (P0) from the published bulk RNA-seq dataset (GSE100934; [56]). (**L**) Graph of the normalized chip signals plotted as gene expression measures from microarray dataset E-MTAB-6868, obtained from human embryonic notochord cells at 7.5–8.5 weeks post-conception (WPC) and fetal NP cells at 12–14 WPC. (**M**,**N**) STRING protein–protein interaction (PPI) analysis, performed using the *Homo sapiens* genes in (**L**) as a reference. Colored circles represent network nodes (proteins); edges represent protein–protein associations, which are not necessarily physical interactions. The sources of the interactions are color coded as follows: blue, interactions derived from a database; fuchsia, experimentally determined interactions; green, text-mining; violet, co-expression; lilac, protein homology. (**M**) PPI-network. (**N**) Unsupervised MCL-clustering identification of four natural clusters (colored in red, yellow, blue, and green) among the nine nodes, based on stochastic flow. Nodes that are part of each cluster are annotated with the same color. Dashed lines indicate inter-cluster edges.

Eight of these genes are described here in connection with Rac1 (Figure 1). The expression of *Rac1* in the *Ciona* notochord in early/mid-tailbud embryos had been previously reported [30], along with its post-metamorphic expression in various structures of juveniles [55] (Table S1); here, we analyzed the expression of this gene at additional developmental stages in order to determine more precisely the duration of its notochord expression and to evaluate the overlap of its expression patterns with that of other signaling molecules. Using WMISH, we detected a distinguishable hybridization signal for *Rac1* in the notochord and mesenchyme of initial tailbud embryos (Figure 1A,B); an initially faint signal in the CNS increased in strength in mid-tailbud embryos, while the notochord expression became undetectable (Figure 1B, inset). Differently from *Rac1*, the expression of *Plcg1* (*phospholipase C gamma 1*), which encodes for a predicted signal transducer, was initially faint in both the notochord and mesenchyme of initial tailbud embryos but increased in both tissues at later stages (Figure 1C). *Grb2* (*growth factor receptor-bound protein 2*) and *Arhgap45* (*Rho GTPase activating protein 45*) are both expressed in initial tailbud embryos in the notochord and mesenchyme (Figure 1D,E); however, the expression of *Grb2* persists at more sustained levels in the notochord of mid-tailbud embryos (Figure 1D, inset) compared to that of *Arhgap45* (Figure 1E, inset). We detected strong expression in the notochord, mesenchyme, and CNS in the case of *Vangl1* (*Van Gogh-like 1*) (Figure 1F); the hybridization signal remained elevated in early tailbud II embryos, which also displayed staining in the trunk endoderm ventral to the sensory vesicle (Figure 1F, inset). *Arl13b* (*ADP ribosylation factor like GTPase 13B*) was also detected in the notochord and mesenchyme of initial tailbuds and persisted in these tissues in mid-tailbuds (Figure 1G). 

*Inpp5b* (*inositol polyphosphate-5-phosphatase B*), is strongly expressed in the notochord and trunk endoderm of mid-tailbud embryos (Figure 1H); earlier during development, expression of this gene is detected in neural precursors of late gastrulae, in addition to the invaginating notochord precursors (Figure 1H, inset). Differently from all other genes described above, *Ptpra* (*protein tyrosine phosphatase receptor A*) is notochord-specific (Figure 1I) and displays a mosaic expression similar to the notochord expression pattern previously described for *Ciona multidom* [31] and *Ctgf* [32]. An even more dishomogeneous expression was observed in the case of *Ywhag*, the gene encoding for 14-3-3 γ protein, whose transcripts were detected only at the anterior and posterior tips of the developing notochord; in addition, this gene was expressed in a small subset of mesenchymal cells that seem to coincide with the B7.7-derived mesenchyme [57] and in small groups of cells of the developing CNS (Figure 1J). *Inpp5b*, *Ptpra*, and *Ywhag* are described in more detail below.

Next, we used published transcriptomic datasets to gauge the evolutionary conservation of the expression of the signaling genes identified in the *Ciona* notochord in the mouse and human notochord and NP cells. As a reference, we included the expression of *Bra/Tbxt*, a well-characterized regulator and marker of the notochord and NP maintenance [58,59,60,61,62]. Expression in mice was analyzed using the GSE100934 bulk RNA-Sequencing (RNA-Seq) dataset generated using mouse notochord cells isolated from E12.5 embryos, and NP cells from newborn mice at post-natal day zero (P0) [56], following the normalization of the gene counts (Figure 1K). The expression of these genes was analyzed in the human notochord (7.5 to 8.5 weeks post-conception, WPC) and newly formed NP (12–14 WPC) using the E-MTAB-6868 microarray dataset and normalized chip signal [63] (Figure 1L).

Next, to determine whether and how these signaling molecules could interact with each other in the notochord, we searched for possible protein–protein interactions (PPIs) among the human orthologs of these 20 *Ciona* genes using the STRING database (STRING-db) [64,65]. This analysis reconstructed a PPI network between these nine genes (Figure 1M) with a PPI enrichment *p*-value = 3.37 × 10^−5^ and 0.63 average local clustering coefficient. No PPIs were found in the case of the remaining eleven genes. The interactions between several of the nine proteins of interest (fuchsia line in Figure 1M,N) have been experimentally determined. Interestingly, GRB2, PLCG1, and RAC1 were in the center of the network, forming key nodes. Unsupervised MCL-clustering generated four natural clusters (Figure 1N). Interestingly, the edges between the main clusters (dashed lines in Figure 1N) were formed by GRB2, PLCG1, and RAC1, supporting the importance of these proteins in the network. Since these results were obtained using human orthologs of *Ciona* proteins, we analyzed the predicted *Ciona* proteins to verify the presence of domains and/or amino acid residues that had been reported to mediate these interactions in other organisms. We verified the possible PPIs between *Ciona* Rac1 and Plcg1, between Rac1 and Grb2, and between Grb2 and Plcg1 using the PEPPI software for protein–protein interaction prediction developed by the Zhang lab (Univ. of Michigan) and available online (https://zhanggroup.org/PEPPI/ (accessed in August 2024) [66]. In support of the possible interaction between Ptpra and Grb2, we found that the most recent predicted amino acid sequence of *Ciona* Ptpra, retrieved from the Ghost database, contains four copies of the YXNX consensus sequence, which has been reported to mediate the interaction with Grb2 [67]. In turn, the predicted *Ciona* Grb2 contains 227 amino acid residues, organized into two SH3 domains flanking a central SH2 domain; this configuration allows the interaction of Grb2 with Ptpra and other proteins (e.g., [68]). The reported interaction between Rac1 and Arhgap45 (also known as Hmha1), which was detected in pull-down assays [69], is likely conserved in *Ciona* as well. Vang-like proteins can associate with Rac1 through their PDZ-binding motif [70], and we verified that the *Ciona* Vangl1 predicted protein contains a conserved C-terminal ETSV amino acid motif that might mediate this interaction [71].

### 2.2. Candidate Components of Diverse Signaling Pathways Are Expressed in the Ciona Notochord

**Figure 2:**
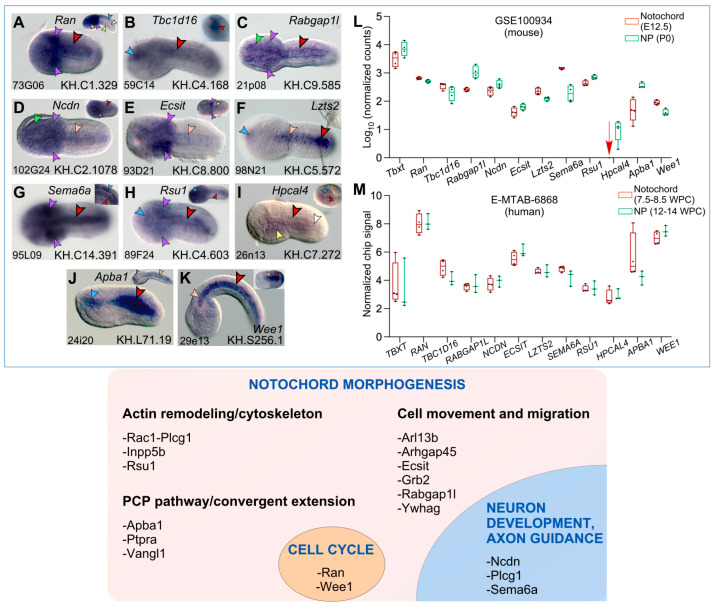
Evolutionarily conserved signaling molecules and their presumptive functions in the *Ciona* notochord. (**A**–**K**) Whole-mount *C. robusta* embryos at stages approximately ranging from late neurula/initial tailbud to late tailbud I [47], hybridized in situ with digoxigenin-labeled antisense RNA probes. Gene names are reported on top of each panel, gene models in the bottom right corners; on the left corner of each panel are reported the ESTs used to synthesize each antisense probe. Insets in (**A**,**B**,**G**,**H**,**J**) display embryos at developmental stages subsequent to those displayed in the main panels; inset in (**B**) shows an optical section of the tail of an older embryo from the same experiment, with the notochord in the center; insets in (**D**,**E**,**G**,**K**) show embryos at earlier stages compared to those in the respective main panels. Arrowheads color code: red, notochord; pink, faint notochord signal; white, unstained notochord; blue, CNS; violet, mesenchyme; yellow, endoderm; green, epidermis. (**L**) Graph of the normalized read counts from the notochord of E12.5 mouse embryos (E12.5) and from NP cells at post-natal day zero (P0) from the published bulk RNA-seq dataset (GSE100934; [56]). (**M**) Graph of the normalized chip signals plotted as gene expression measures from microarray dataset E-MTAB-6868 [63], obtained from human embryonic notochord cells at 7.5–8.5 weeks post-conception (WPC) and fetal NP cells at 12–14 WPC. Bottom panel: summary of the presumptive functions of 17 of the genes identified in this study, inferred from studies carried out in other organisms (individually cited in the main text).

As shown in Figure 2, this study also identified eleven additional *Ciona* notochord genes encoding for presumed signaling molecules. The predicted protein of *Ran* (*Ras-related nuclear protein*) is a small GTP-binding protein; we detected expression of this gene in the notochord, along with strong expression in the mesenchyme (Figure 2A) of initial tailbuds; the hybridization signal was lost in notochord cells of early tailbuds (inset in Figure 2A).

*Tbc1d16*, which is predicted to encode for an activator of GTPases of the Rab family, is expressed in the developing sensory vesicle and, more faintly, in the notochord of early tailbud embryos (Figure 2B); the expression in the notochord increases in older embryos (mid-tailbud), as demonstrated by the analysis of different focal planes of images of the tail (inset in Figure 2B). Another gene encoding a Rab-GTPase-activating protein, *Rabgap1l*, is expressed in the notochord and throughout the trunk region in tailbud embryos (Figure 2C).

Transcripts for a putative neurochondrin (ncdn; also known as norbin in mouse), a predicted GPCR adaptor protein, are detected in the notochord and trunk of mid-tailbud embryos (Figure 2D); however, the expression of this gene appears stronger in initial tailbuds (Figure 2D, inset).

*Ecsit* (evolutionarily conserved signaling intermediate in Toll pathways) encodes for an adaptor protein that is considered to act at the intersection of the Toll and BMP signaling pathways [72]. We detected weak expression of this gene in the notochord of initial and mid-tailbuds, while the expression in mesenchymal cells remained consistently higher (Figure 2E).

The predicted product of *Lzts2* (*Leucine-zipper tumor suppressor 2*) is a β-catenin binding protein that regulates the nuclear localization of β-catenin [73]; this gene is expressed in a small region of the anterior sensory vesicle and displays a gradated expression in the notochord (Figure 2F and see below).

Semaphorin 6a (Sema6a) is predicted to function as a receptor involved in repulsive axon guidance (e.g., [74]); our WMISH results indicate that, in addition to *Sema3A* [75], *Ciona Sema6a* is expressed in the notochord, starting around the initial tailbud II stage (Figure 2G); before this stage, expression is detected in the developing nerve cord and in the mesenchyme (Figure 2G, inset). Analysis of the published single-cell RNA-Seq (scRNA-Seq) data available for two *Ciona* genes annotated as *plexin A* suggests that at least one of them (gene model: KH.C7.348) is expressed in notochord cells (Figure S1), which suggests the possibility that semaphorin–plexin complexes might be formed during notochord morphogenesis.

The expression of *Rsu1* (Ras suppressor protein 1) is first detected in notochord cells around late neurulation; in tailbuds, additional hybridization signals are visible in the mesenchyme and part of the CNS (Figure 2H); the latter territory widens in mid-tailbud embryos, and the overall staining becomes more intense (Figure 2H and inset). In other organisms, Rsu1 interacts with Pinch1, a LIM-domain protein, to form a cytoplasmic complex that colocalizes with integrin-rich focal adhesions [76]; through BLAST searches, we identified a *Pinch1* ortholog in *Ciona* (gene model: KH.C4.177), and by analyzing published scRNA-Seq data [77], we found evidence that this gene is also expressed in notochord cells (Figure S1).

Hippocalcin-like 4 (Hpcal4), a predicted calcium-binding protein, is mainly expressed in the notochord and trunk endoderm of initial tailbuds (Figure 2I), although at the gastrula stage it is also detected in neural precursor cells (Figure 2I, inset).

*Apba1* (amyloid beta precursor protein binding, also abbreviated as *X11*, *Mint1*) displays strong expression in the intercalating notochord cells of initial tailbuds, and discernible hybridization signal in a small region of the developing sensory vesicle (Figure 2J); in late tailbuds, the notochord expression appears considerably reduced (Figure 2J, inset). Lastly, the *Ciona* gene encoding for a protein orthologous to the mitosis inhibitor protein kinase Wee1 is expressed predominantly in notochord cells (Figure 2K), beginning around late neurulation (Figure 2K, inset). Similarly to that of *Lzts2*, the expression of *Wee1* in notochord cells resembles an anterior–posterior gradient (Figure 2K and results shown below).

The majority of these genes were represented in the mouse notochord and NP (Figure 2L), with two exceptions: *Arhgap45* and *Hpcal4. Arhgap45* was not detected in either the mouse or the human dataset. In the case of *Hpcal4*, only one replicate at E12.5 showed one read, while the other three replicates showed zero reads, indicating that this gene may be expressed transiently and/or at low levels, if at all, in the mouse notochord (red arrow in Figure 2L). On the other hand, *HPCAL4* was represented in the human notochord and NP cells, with expression levels comparable to those of *BRA/TBXT* (Figure 2M).

### 2.3. Dishomogeneous Expression of Signaling Molecules in the Ciona Notochord

**Figure 3:**
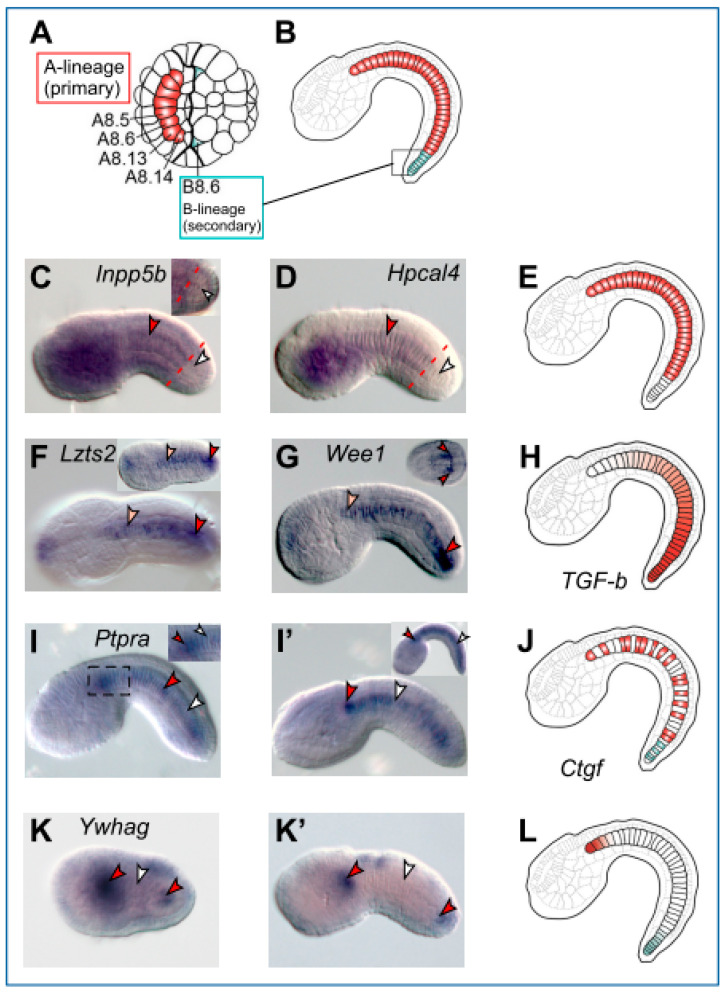
Signaling molecules heterogeneously expressed in the *Ciona* notochord. (**A**,**B**,**E**,**H**,**J**,**L**) Drawings of *Ciona* embryos at the 110-cell stage (**A**) and at the late tailbud stage (**B**,**E**,**H**,**J**,**L**), illustrating (**A**) the A- and B-lineage notochord precursors and (**B**) their daughter cells, which form the primary and secondary notochord, respectively, and the diverse expression patterns that have been observed and/or previously reported for notochord genes (**E**,**H**,**J**,**L**). (**C**,**D**) *Inpp5b* and *Hpcal4* are expressed predominantly in the primary notochord and appear excluded from the secondary notochord (**E**); inset in (**C**) shows the tip of the tail of a different embryo, which, in addition to the image in the main panel, further illustrates the difference in hybridization signal between the primary and secondary notochord. In (**C**,**D**), the boundary between these two groups of notochord cells is approximately delineated by a red dashed line. (**F**,**G**) *Lzts2* and *Wee1* display gradated anterior–posterior expression, similarly to *TGF-β* [32] (**H**). Insets in (**F**,**G**) show embryos at earlier developmental stages. (**I**,**I’**) *Ptpra* is expressed mosaically in a lineage-independent fashion, both at early (**I**) and late (**I’**) developmental stages, similarly to *multidom* [31]. (**J**). Inset in (**I**) shows a close-up of the region boxed in black in the main panel; inset in (**I’**) shows a late tailbud. (**K**,**K’**) *Ywhag* is expressed exclusively at the anterior and posterior tips of the notochord at early (**K**) and late (**K’**,**L**) stages. Gene models and ESTs are reported in Figure 1 and Figure 2 and in Table S1. Arrowheads color code: red, notochord staining; pink, weak notochord staining; white, absence of notochord staining.

Six of the notochord genes analyzed in this study display dishomogeneous expression in the developing *Ciona* notochord. The *Ciona* notochord is derived from two separate groups of precursor blastomeres (Figure 3A) and is classically annotated as either “primary” (the anterior-most 32 cells) or “secondary” (the posterior-most eight cells) (Figure 3B). We found that two genes, *Inpp5b* and *Hpcal4*, are exclusively expressed in the primary notochord and are excluded from the secondary (Figure 3C–E; see also Figure 1H and Figure 2I). In addition to this clear-cut lineage-specific expression, we detected a gradient-like expression in the case of *Lzts2* and *Wee1* (Figure 3F,G) (see also Figure 2F,K), which resembles the expression previously reported for *TGF-β* [32] (Figure 3H). The expression of *Ptpra* (Figure 3I,I’; see also Figure 1I) mirrors the lineage-independent mosaic pattern that our group had previously reported for *multidom* [31] and has also been reported in the case of *Ctgf* (Figure 3J; [32]). Lastly, the expression of *Ywhag* was characteristically localized to the anterior-most and posterior-most tips of the notochord and was excluded from the remainder of the notochord cells at the stages that we analyzed (Figure 3K,L; see also Figure 1J).

### 2.4. Identification and Functional Characterization of a Notochord Enhancer Associated with Cr-TGF-β

In *Ciona robusta,* the transforming growth factor beta gene (*Cr-TGF-β*; gene model: KH.C3.724) encodes for a predicted protein of 439 amino acid residues; BLAST searches indicate that its sequence exhibits 35%, 40%, and 36% similarity to the three human isoforms of the secreted ligands of the TGF-β superfamily: TGF-β1, TGF-β2, and TGF-β3, respectively. As previously reported, *Cr-TGF-β* transcripts are first detected by the early neurula stage in notochord cells and persist in the notochord until the tailbud stages [32]. Noticeably, the hybridization signal appears stronger in the posterior notochord than in its anterior region; this gradated expression pattern is initially lineage-independent, but in late tailbuds appears restricted to the posterior-most cells of the notochord, almost exclusively to the eight cells that compose the secondary notochord [6,32].

**Figure 4:**
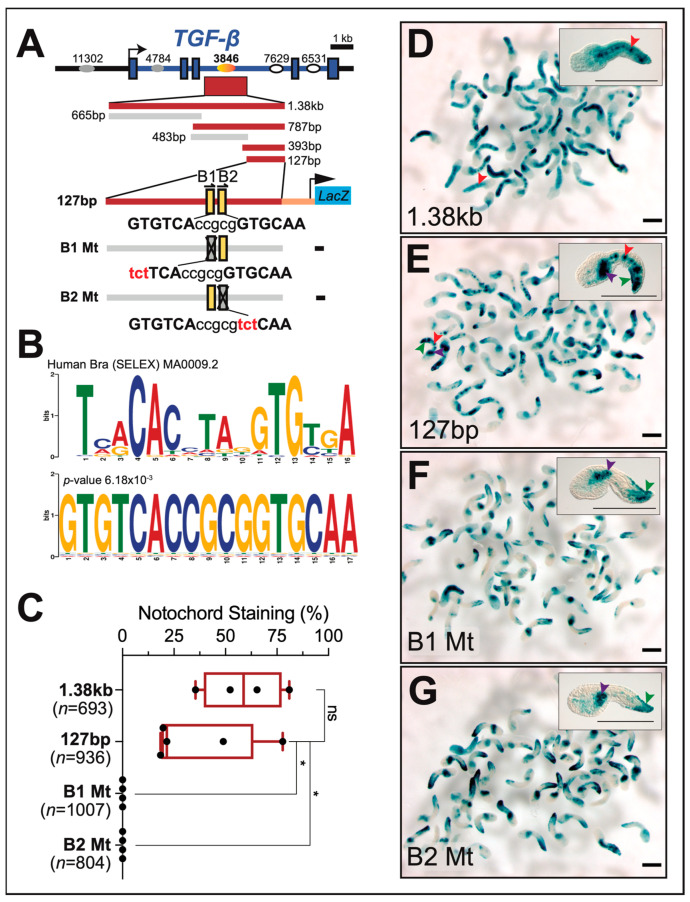
Identification and functional characterization of the *Cr-TGF-β* notochord CRM. Identification of the putative TF binding sites necessary for the transcriptional activity of the *Cr-TGF-β* notochord enhancer, achieved through sequence-unbiased truncations and site-directed mutations. (**A**) Schematic representation of the location of the *Cr-TGF-β* notochord enhancer region (red horizontal bar), which maps within intron 3 and encompasses ATAC-Seq peak 3846 (orange oval) [78]. Grey ovals symbolize ATAC-Seq peaks that were inactive when tested in vivo; white ovals represent ATAC-Seq peaks that were not tested. The “-” signs indicate a complete absence of notochord activity. Putative Ci-Bra binding sites are depicted as yellow vertical bars, numbered as B1 and B2; small arrows on top of each bar indicate the orientation of each binding site. (**B**) Motif–motif similarities between the functional Ci-Bra binding site in the *Cr-TGF-β* notochord CRM and the human BRA/TBXT consensus sequence identified by SELEX assays. Sequence comparison analysis was performed using Tomtom software Version 5.5.7 and the databases JASPAR Vertebrates (Version 2024) and Uniprobe [79]. (**C**) Quantification of notochord activity in embryos electroporated with constructs containing single mutations in the Ci-Bra binding sites, (B1) and (B2), as detected by X-Gal staining. The total number of X-Gal-stained embryos (*n*) analyzed per experiment is reported underneath the *x*-axis. Data represent 25th to 75th percentiles (bounds of box), median (center line) ± min to max (whiskers); asterisk indicates *p* < 0.05; ns, non-significant; two-sided Student’s *t* test (*n* ≥ 3 biologically independent samples per category). (**D**–**G**) Low-magnification group photomicrographs of mid-tailbud embryos electroporated in parallel with either the 1.38 kb *Cr-TGF-β* notochord CRM (**D**), the 127 bp CRM (**E**), or with constructs carrying single mutations (**F**,**G**) in the Ci-Bra binding sites, respectively. Mutated sequences are in lower case and colored in red. The mutations were as follows: B1 Mt: GTGTCA to tctTCA; B2 Mt: GTGCAA to tctCAA. Insets show higher magnification photomicrographs of representative embryos stained by X-Gal; arrowheads indicate the embryonic tissues stained by each construct: red, notochord; purple, mesenchyme; green, epidermis. Scale bars: 200 μm.

An analysis of the chromatin landscape of the *Cr-TGF-β* genomic locus highlighted five regions with an open chromatin conformation (ATAC-Seq peaks; [78], one located upstream of the coding region and the other four dispersed within intronic regions (Figure 4). Three of the five ATAC-Seq peaks (peak 11302, located in the 5′ region, peak 4784, located in intron 1, and peak 3846, in intron 3; [78]) were cloned and tested in vivo by electroporation in *Ciona* zygotes. We found that a 1.38 kb regulatory sequence encompassing the 254 bp ATAC-Seq peak 3846 drives strong expression of the *LacZ* reporter in the notochord cells of both lineages (Figure 4A). Through serial truncations, this sequence was narrowed down to 127 bp; sequence analysis of this 127 bp region revealed the presence of two adjacent putative Ci-Bra binding sites in tandem orientation; a comparison between these sites and the consensus binding site for human BRA/TBXT is shown in Figure 4B. Individual mutations in either Ci-Bra binding site eliminated the activity of this 127 bp region, suggesting that both Ci-Bra sites are indispensable for the activity of the *Cr-TGF-β* notochord CRM (Figure 4A,C–G). These findings are corroborated by the reported occupancy of the *Cr-TGF-β* locus by a Ci-Bra-GFP fusion protein in ChIP-chip assays [52] and by the response of *Cr-TGF-β* to the ectopic expression of *Ci-Bra* in misexpression assays [54].

**Figure 5:**
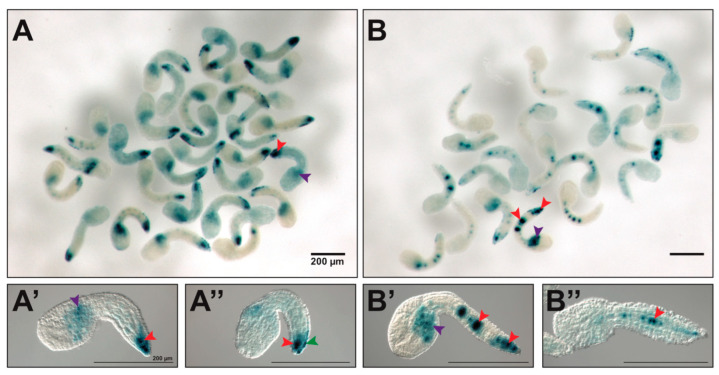
Regionalized activity of the *Cr-TGF-β* notochord CRM. To quantify the frequency of embryos with primary, secondary, or primary/secondary notochord staining, *Ciona* zygotes were electroporated with 75 µg of the 1.38 kb *TGF-β* notochord enhancer region (Figure 4A). After electroporation, embryos were cultured during the day at 21.5 °C and fixed for ~8 hrs after fertilization. X-Gal staining was carried out at 37 °C for ~20 h In a representative experiment, of the total embryos analyzed (*n* = 206), 73 embryos displayed notochord staining (35.4%). Of the 73 embryos with notochord staining, 19 showed only primary notochord staining (26%), 51 showed only secondary notochord staining (~70%), and only 3 displayed staining in both the primary and secondary notochord (~4%). (**A**) Low-magnification photomicrograph of a group of *Ciona* embryos showing staining only in the secondary notochord, selected from different replicates of the experiment described above. (**B**) Low-magnification photomicrograph of a group of *Ciona* embryos showing staining in both the primary and secondary notochord, selected from different replicates of the experiment described above. (**A’**,**A”**) Higher magnification views of embryos with staining in the secondary notochord. (**B’**,**B”**) Higher magnification views of embryos with staining in both the primary and secondary notochord. Arrowheads color code: notochord (red), mesenchyme (purple), epidermis (green). Scale bars: 200 μm.

In an effort to assess whether the notochord enhancer region that we identified was able to recapitulate the nuanced signal that was observed by WMISH for *Cr-TGF-β* [6,32], we used varying amounts of plasmid DNA and different X-Gal staining conditions, closely monitoring the notochord cells as the staining progressed. These additional experiments, reported in Figure 5, showed that, unlike what was found for notochord enhancers associated with homogeneously expressed genes (e.g., [80]), a considerable fraction (~70%) of the transgenic embryos carrying either the 1.38 kb or the 127-bp enhancer region displayed staining exclusively in the secondary notochord (Figure 5A–A”), even though they are able to direct reporter gene expression in both the primary and secondary notochord (Figure 5B–B”).

### 2.5. Identification and Functional Characterization of a Notochord Enhancer Associated with Cr-Ctgf

*Cr-Ctgf* is first detected in notochord cells at mid-tailbud I (stage 21, [47]), after intercalation is complete, and persists until late tailbud I (stage 23) [47]. Even though this gene is expressed in a stochastic manner, similar to the previously described *multidom* [31], its expression becomes increasingly more homogeneous as tail elongation proceeds, and in late tailbuds, can be observed in most notochord cells [32], while *multidom* expression remains highly mosaic [31].

**Figure 6:**
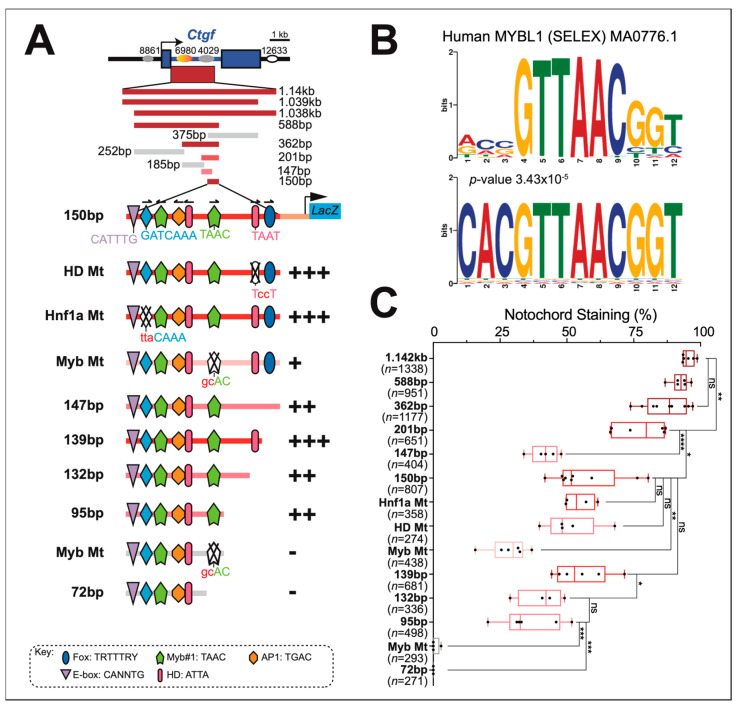
Identification and functional characterization of the *Cr-Ctgf* notochord CRM. Identification of the minimal sequences necessary for the transcriptional activity of the *Cr-Ctgf* notochord enhancer in reporter assays, through sequence-unbiased truncations and site-directed mutations. (**A**) Schematic representation of the location of the *Cr-Ctgf* notochord enhancer (red horizontal bar), which lies within intron 1 and encompasses ATAC-Seq peaks 6980 and 4029 (ovals) [78]. The ATAC-Seq peak 6980 (orange oval) is included in the notochord enhancer; tested ATAC-Seq peaks with no activity are depicted as gray-colored ovals; a white oval denotes an untested ATAC-Seq peak. The “+” and “−” signs are used to show the presence or absence of notochord activity, respectively. Putative TF binding sites are symbolized by geometric shapes of different colors; small arrows on top of each bar indicate the orientation of each binding site. Mutated sequences are in lower case and colored in red. The mutations were as follows: HD Mt: TAAT to TccT; Hnf1a Mt: GATCAAA to ttaCAAA; Myb Mt: TAAC to gcAC. (**B**) Motif–motif similarities between the functional Ci-Bra binding site in the *Cr-Ctgf* notochord CRM and the human MYBL1 consensus sequence identified by SELEX assays. Sequence comparison was performed as described in Fig. 4. (**C**) Quantification of the results of the truncation/mutation analysis of the *Cr-Ctgf* notochord CRM, determined by X-Gal staining. The total number of X-Gal-stained embryos (*n*) analyzed per experiment is reported underneath the *x*-axis. Data represent 25th to 75th percentiles (bounds of box), median (center line) ± min to max (whiskers); * *p* < 0.05, ** *p* < 0.01, *** *p* < 0.001, **** *p* < 0.0001, ns, non-significant; two-sided Student’s *t* test (n ≥ 3 independent samples per category). The intensity of the notochord staining elicited by each truncation and mutation is reported using different shades of red and pink, with red indicating full intensity and lighter red/pink indicating faint notochord staining.

**Figure 7:**
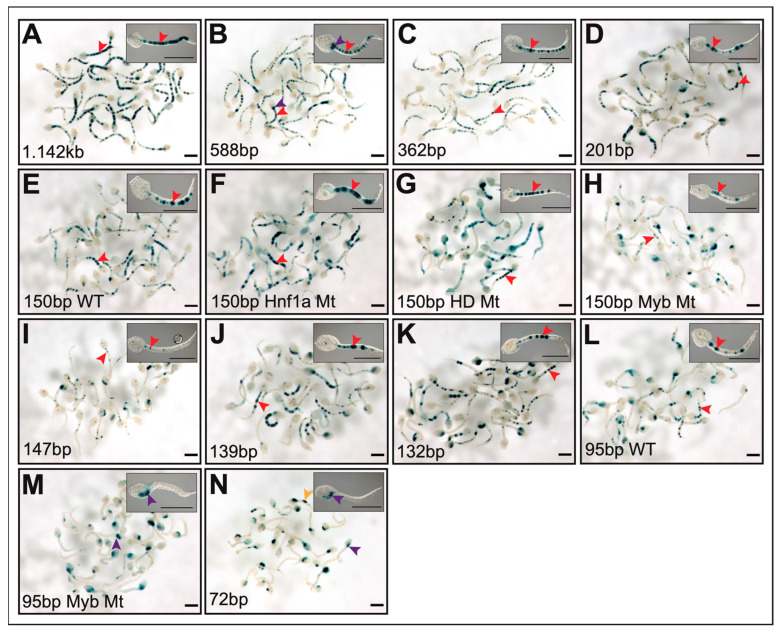
In vivo activity of different truncated and mutant versions of the *Cr-Ctgf* notochord enhancer region. (**A**–**N**) Low-magnification group photomicrographs of mid-tailbud embryos electroporated in parallel with the transgenes indicated in the lower left corner of each panel and stained with X-Gal. Insets in (**A**–**N**) show high-magnification photomicrographs of the representative embryos from each experiment. Arrowheads color code: red, notochord; purple, mesenchyme; orange, muscle. Scale bars: 200 μm.

Published data on chromatin accessibility of the *Cr-Ctgf* genomic locus [78] indicated the presence of open-chromatin regions in the 5′-upstream and first intron of *Cr-Ctgf*. We cloned and tested in vivo a 1.142-kb genomic fragment encompassing ATAC-Seq peaks 6980 and 4029, and found that it directed reproducible reporter gene expression in notochord cells (Figure 6A). Through sequence-unbiased serial truncations, this notochord enhancer was narrowed to a 362-bp region that displays activity comparable to that of the 1.142-kb fragment (~90% notochord staining; Figure 6A,C). Further truncation of the 362-bp region to 150 bp led to the reduction of notochord activity to ~60% (Figure 6A,C). Interestingly, this 150-bp notochord CRM is devoid of canonical binding sites for Ci-Bra (TNNCAC; [81]), but contains putative binding sites for TFs of the homeodomain (HD), bHLH, Myb, AP1, and Fox families, which have been previously shown to be necessary for the activity of different subclasses of *Ciona* notochord CRMs (Figure 6A,B; [9,11,82]). In the 150-bp sequence, site-directed mutations in one of the predicted binding sites for Myb-like TFs (Figure 6B) significantly reduced the notochord activity (~25% notochord staining; Figure 6C). Both the mutation of this site in a 95-bp construct and its truncation, which narrowed the CRM to a 72 bp region, resulted in a complete loss of notochord activity (Figure 6A,C). To further evaluate whether other TF binding sites contributed to the notochord activity, we tested serial truncations of the 3′-end of the 150-bp construct, spanning 139 bp, and 132 bp, respectively. Truncating the 3′-end of the notochord CRM from 150 bp to 139 bp eliminated a putative Fox binding site, with no detectable effect on the notochord activity (Figure 6A,C). These results indicate that the *Cr-Ctfg* notochord CRM mainly, although not exclusively, relies on a binding site for a TF of the Myb family. Additional binding sites for minor activators seem to be interspersed along the 1.142-kb *Cr-Ctfg* genomic fragment and also influence the overall intensity of the notochord staining. These findings are illustrated in the graph in Figure 6C, where in addition to the quantification of the respective staining percentages, the qualitative intensity of the staining elicited by each truncation and mutation is depicted through the use of different shades of red and pink. Figure 7 shows low-magnification group photos of transgenic embryos carrying the 1.142-kb *Cr-Ctfg* genomic fragment (Figure 7A) and the truncated and mutant constructs derived from it (Figure 7B–N), along with high-magnification insets that display in detail the intensity of the notochord staining that each transgene is able to induce.

## 3. Discussion

### 3.1. Expression of Components of the Rac1 Signaling Pathway in the Ciona Notochord

Rac1 is a member of the Rho family, which constitutes a large branch of the Ras superfamily of GTPases; these proteins are primarily involved in the control of the dynamic remodeling of the cytoskeleton (e.g., [83]). As such, Rac1 participates in a multitude of vital cellular processes, and also in carcinogenesis and metastasis [84]. In the *Xenopus* notochord, Rac and Rho GTPases are the main mediators of convergent extension movements, acting downstream of the Wnt-planar cell polarity (Wnt-PCP) pathway [85].

Plcg1 is one of the isoforms of phosphoinositide-specific phospholipase C, a signaling molecule involved in extracellular matrix (ECM) synthesis; in mice lacking *plcg1*, hematopoiesis is severely impaired, along with endothelial development [86]. Interestingly, the removal of this gene in neuronal progenitors affects axon guidance, which suggests that Plcg1 acts as a mediator of the netrin/DCC signaling pathway [87]; the concomitant expression of *netrin* and *Plcg1* in the *Ciona* notochord suggests that in addition to being part of the Rac1 pathway, Plcg1 might be mediating the function of netrin as well.

Another candidate interactor of Rac1, Grb2, is an adaptor protein characterized by the presence of a Src homology type 2 (SH2) domain and two Src homology type 3 (SH3) domains, and was first described in *C. elegans* [88]. Grb2 has also been associated with cell motility and the cell cycle, and its roles in metastasis and hematopoiesis have been elucidated [89]. In addition, in human hematopoietic cells, this adaptor interacts with STAT5; in *Ciona*, the expression of a *Stat5* ortholog in the developing notochord [8] suggests that this interaction might be occurring during notochord formation. Another gene expressed in hematopoietic cells is *Arhgap45*, which encodes a Rac-GAP also known as Hmha1; in mice, Arhgap45 controls the migration of B- and T-lymphocytes into lymph nodes [90]. The expression in the *Ciona* notochord of genes that in vertebrates are involved in hematopoiesis is intriguing, since several *Ciona* genes expressed in the notochord have been reported to switch to circulating hematocytes after metamorphosis, at the time of the disappearance of the notochord [55].

Together with another transmembrane protein, Frizzled, and with two cytoplasmic proteins, Prickle (Pk) and Dishevelled (Dvl), Van Gogh/Strabismus (Vang/Stbm) proteins are central elements of the PCP pathway; studies conducted primarily in *Ciona savignyi* have shown that the localization of Pk, Dvl, and Vang/Stbm to the anterior poles of the notochord cells, which occurs around mid-tailbud (stages 21/22), is responsible for the polarized cytoskeletal changes required for the subsequent steps of notochord morphogenesis [91,92]; in *Ciona robusta*, Vangl1 (KH.C4.173) morphants display a “disorganized body plan” phenotype [93,94].

Arl13b plays an evolutionarily conserved role in ciliary movements and transport in ascidians [95], and participates in Sonic hedgehog signaling in other systems [96]; mutations in this gene lead to Joubert syndrome in humans, and to a related mutant phenotype in zebrafish [97]. In addition, this Arf-like protein has been shown to be required for cell migration [98]; its role in notochord development is still unknown, and its expression in the intercalating notochord cells of *Ciona* suggests that it might contribute to notochord cell movements.

The phosphatase Inpp5b has been described as a regulator of actin remodeling, able to hydrolyze PIP2 in the plasma membrane, causing the disassembly of cytoskeletal F-actin, likely in conjunction with additional factors [99]. As such, it might counteract the function of Rac1-Plcg1 complexes, which promote F-actin formation (e.g., [100]).

### 3.2. Signaling Molecules Potentially Involved in Cell Division, Cell Migration, and Axon Guidance

Ran encodes for a small GTP-binding protein; in *Xenopus*, one of its orthologs is expressed in the CNS, neural crest, sensory organs, and mesenchyme, but is not detected in the notochord and somites [101]. Nevertheless, the microarray data from human embryonic and fetal notochord cells [63] analyzed in this study suggest that Ran is expressed in the human notochord. Among the multiple functions affected by Ran and its various interacting proteins in other systems are the control of nucleus-cytoplasm transport, and its participation in cell cycle progression through its contribution to the formation of the mitotic spindle [102].

*Neurochondrin* is expressed in the NP cells of rat intervertebral discs [103]; while its specific function in this structure is still unclear, the role of this Leucine-rich GPCR adaptor protein in human neuronal growth and neural development has been ascertained [104].

Originally classified on the basis of their characteristic axon-repellent activity, semaphorins have more recently been highlighted as regulators of cell cohesion and motility that play critical roles in the normal development of various tissues and in tumorigenesis [105,106]. In vertebrates, the notochord is a crucial source of axon guidance molecules that repel axons emerging from the dorsal root ganglia [107], the sympathetic nervous system, and the retina [108]. These molecules include not only semaphorins emanating directly from the notochord cells, but also notochord-secreted chondroitin sulfate proteoglycans (CSPGs), which are localized to the notochordal sheath [107]. Of note, the notochord of *Ciona* and other tunicates also expresses the axon-attractant *netrin* [109,110] and its morpholino-mediated knockdown affects notochord intercalation [47]. A comparison of the expression pattern of *Ciona netrin* [109] with that of *Sema6a*, reported here, indicates that *netrin* is expressed early during development in neural and notochord precursor cells, and persists, mainly in the notochord, in the tailbud stages [109], while *Sema6a* is expressed discontinuously in cells of the developing neural tube at the time of neurulation and following stages, and is expressed transiently and at low levels in notochord cells. Another *semaphorin* gene, *Sema3a* (gene model: KH.C10.17), is expressed in the *Ciona* notochord, predominantly, although not exclusively, at the tips of the notochord [75]. This suggests that the dynamic expression of these guidance molecules might direct the growth of axonal tracts adjacent to the notochord. Together, these results cement the notion that, in addition to its evident supporting role, the ascidian notochord provides patterning signals to adjacent cells in the developing CNS.

Rsu1 is localized to the cell-ECM adhesions and modulates the Ras signaling pathway by suppressing Ras-dependent tumorigenesis and metastasis [111]. This function is dependent upon the interaction of Rsu1 with Pinch1 (also known as LIMS1), a LIM-type Zinc-finger protein that directly binds cytoplasmic F-actin, bridging the cytoskeleton to receptor complexes on the cell surface [76]. The concomitant expression of Rsu1 and Pinch1 suggests that Rsu1-Pinch1 complexes might be present in the developing *Ciona* notochord, where they might control cell adhesion and migration.

### 3.3. Genes Associated with AD Pathogenesis Are Expressed in the Ciona Notochord

Proteins of the Apba1/X11/Mint1 family function as adaptors and are able to bind the amyloid precursor protein (App), the precursor of amyloid beta peptide (Abeta); by doing that, they prevent the proteolytic step leading to Abeta production and consequent accumulation in neuritic plaques, a process associated with the aetiogenesis of Alzheimer’s disease (AD) [112]. Remarkably, a bioinformatic study of the *Ciona* genome has shown that this organism possesses orthologs of genes associated with AD, including *App*, *presenilin*, and *nicastrin*, among others; these findings, along with the reported ability of ascidian larvae to process human APP695 and form amyloid-like plaques, argue in favor of the use of ascidian embryos for studies of basic mechanisms of AD pathogenesis and response to therapeutics [113]. The expression of *App* genes in the notochord has been reported in chicks, in the case of *Apba2*, a gene related to *Apba1* [114], and in zebrafish, where morpholino-mediated knockdowns of APPa and APPb proteins induce defective convergence-extension and dose-dependent notochord phenotypes [115]. Our previous studies have shown that, in addition to *Apba1*, two other *Ciona* orthologs of genes associated with AD, *nicastrin* (KH.C1.1147) and *neprilysin* (KH.C12.669), are expressed in the developing notochord (Jose’-Edwards et al., 2013). Interestingly, a study carried out in a *C. elegans* AD model [116] determined that *Tbc1d16*, which encodes a predicted Rab GTPase-activating protein, is one of the differentially expressed genes shared with the human AD brain transcriptome [117]. Another gene that is potentially associated with AD is the human ortholog of *Rabgap1l*, (also known as KIAA0471) [118], which is involved in endolysosomal trafficking in migrating fibroblasts [119]. *Ecsit* is required for mesoderm development in mouse embryos; knockdown experiments have shown that in its absence, both Bmp and Toll signaling pathways are inhibited [120]; recent reports have linked ECSIT to AD pathogenesis [121].

Remarkably, in the colonial ascidian *Botryllus schlosseri*, amyloid fibrils are produced by immunocytes and are involved in the immune response [122]; however, the function of the components of the amyloidogenetic pathway in notochord formation remains to be explored. In murine cell cultures, fibrillar Abeta deposits associate with perlecan, a heparan sulfate proteoglycan (HPSG) [123]; the expression of *perlecan* (gene model: KH.C3.21; [124]) in the *Ciona* notochord suggests the hypothesis that amyloid-HPSG fibrils might form in the ECM that composes the notochord sheath.

### 3.4. Differentially Deployed Signaling Molecules Specify and Sculpt the Ciona Notochord

Previous reports have demonstrated the role of evolutionarily conserved pathways, FGF/MAPK/MEK/ERK, β-catenin/TCF/LEF, Nodal, and Delta/Notch/Su(H) in the induction of the notochord fate in the ascidian embryo [15,17,18,20,125,126,127,128]. In particular, compelling experimental results indicate that the β-catenin/TCF/LEF pathway, through one of its effectors, the TF FoxD, activates *Ci-Bra* expression in the primary notochord, while the Delta/Notch/Su(H) pathway is responsible for the transcriptional control of this crucial activator of notochord morphogenesis in the secondary notochord cells [16,126]. In addition to these reports, the present study and a few previous ones uncovered the lineage-specific, mosaic, gradated, or tip-specific expression of an increasing number of notochord genes [31,32,33,34]. Specifically, previously reported genes encoding for signaling molecules that are expressed in the developing notochord include *Rxfp2* (*relaxin family peptide receptor 2*; gene model: KH.C8.378), a putative member of the GPCR family, which is predominantly expressed in the primary notochord [33], *RhoB* (*Ras homolog family member B*; gene model: KH.C1.129; previously *RhoDF*), which displays mosaic notochord expression and is required for the protrusive activity of notochord cells [34], and two more genes, *Gnai1* (*G protein subunit alpha i2*; gene model: KH.C2.872) and *Sema3a* (gene model: KH.C10.17), which are both expressed more intensely, although not exclusively, at the anterior and posterior tips of the notochord [33,75].

This study uncovered that Inpp5b and Hpcal4 are both expressed exclusively in the primary notochord. While Inpp5b is a well-characterized regulator of actin remodeling during development, Hpcal4 is a calcium sensor whose role in development is still unclear, and whose expression in the notochord and/or its derivatives had not been previously described. In mice, *Hpcal4* is expressed in excitatory interneurons of the dorsal horn of the spinal cord, where it plays a minor role in the processing of pain and itch stimuli [129]. 

Similarly to *TGF-β*, also *Lzts2* and *Wee1* are expressed in an anterior–posterior gradient. Lzts2 is a regulator of the Wnt/β-catenin signaling pathway and, in zebrafish embryos, it modulates cell movements by reducing the rate of β-catenin nuclear import [73,130], while Wee1, a nuclear tyrosine kinase, is part of the cell-cycle checkpoint molecular machinery. Wee1 exerts its regulatory role by binding cyclin-dependent kinase (Cdk1) and limiting its mitosis-initiating activity in response to DNA damage; as such, it is an effector upon which different transduction pathways converge in response to DNA damage, and a preferred therapeutic target for a variety of cancers [131]. Of note, Wee1 is an inhibitor of the G2/M transition [132]; our results indicate that its expression in the *Ciona* notochord begins around the time of the last cell division of the notochord precursors. These findings suggest that Wee1 might work in addition to Cdkn1.b, a previously characterized cell-cycle regulator expressed in the notochord and other tissues [133], in maintaining a fixed number of post-mitotic notochord cells in the *Ciona* embryo. Even though neither the ChIP-chip experiments nor other assays have suggested a regulatory relationship between Wee1 and Ci-Bra, our unpublished results indicate that Wee1 responds to the ectopic expression of Ci-Bra in heterologous tissues, suggesting that Ci-Bra might control, either directly or indirectly, the expression of this checkpoint regulator.

We were unable to retrieve information on the notochord-specific function of *Ptpra*, which is expressed in a mosaic pattern, similarly to *Ctgf*; however, *ptpra-/-* zebrafish embryos display defects in convergence-extension and a shorter body axis compared to controls, even though *ptpra* expression has not been reported in the notochord of this organism [134].

Lastly, Ywhag is expressed predominantly at the tips of the notochord. Ywha/14-3-3 proteins appear as central nodes in different signaling pathways; in particular, Ywhag (a tyrosine 3-monooxygenase/tryptophan 5-monooxygenase activation protein gamma) is considered an initiator of the epithelial–mesenchymal transition (EMT), and, therefore, a promoter of cell migration and metastasis [135]. While the notochord-specific function of Ywhag remains to be ascertained, another *Ciona* 14-3-3 protein, 14-3-3 ε-a, has been shown to be required, together with its binding partner ERM (ezrin-radixin-moesin), for the flow of lumen-associated components from the basal to the apical domain of notochord cells during tubulogenesis [136].

### 3.5. Dishomogeneity of Gene Expression in the Notochord: Insights from the Analysis of Notochord Cis-Regulatory Modules Associated with Ciona Robusta TGF-β and Ctgf

The approach used in this study, consisting of the analysis of gene expression by WMISH across multiple developmental stages on a large number of embryos (>100 embryos per stage), has allowed the identification of transient and/or dishomogeneous patterns of gene expression in the notochord of *Ciona*. The results presented here indicate that this experimental approach remains a valid complement to RNA-Seq studies and allows a granular spatio-temporal resolution of gene expression dynamics. In an attempt to shed light on the molecular underpinnings of discontinuous expression patterns, we focused on *Ciona TGF-β* and *Ctgf*, due to the conserved roles of these signaling pathways in notochord formation across vertebrates, and pursued the isolation of notochord enhancer regions associated with these genomic loci, along with the identification of the minimal sequences required for their respective activity in notochord cells.

Our findings on the *TGF-β* notochord enhancer region revealed that Ci-Bra controls transcription of this gene, either by directly binding its notochord CRM or via Tbx2/3, which has a similar binding site and acts as a transcriptional intermediary of Ci-Bra [137]. The results of site-directed mutagenesis experiments indicate that the ablation of either Ci-Bra binding site causes a complete loss of notochord activity; however, the predominance of the secondary notochord staining suggests that this main Ci-Bra-downstream “on/off” switch is likely complemented by additional activators that augment expression specifically in the posterior regions of the notochord, thus generating a gradated pattern. Of note, a notochord enhancer region from a gene exclusively expressed in the posterior-most region of the *Ciona* notochord, related to vertebrate *fibulin-5* (gene model: KH.C11.331), has been isolated and characterized in vivo [138]; this enhancer contains binding sites for widespread notochord activators, in addition to a distinct silencer region that narrows the expression to the secondary notochord [138].

Unlike *TGF-β*, the *Ctgf* notochord CRM is devoid of Ci-Bra binding sites that might act as “on/off” switches, and although it mainly relies on a putative Myb-like binding site, the mutation of this sequence, which eliminates notochord activity when introduced within the 95-bp enhancer fragment, it only weakens the 150-bp notochord enhancer but fails to inactivate it completely. Together with the declining intensity of the staining elicited by increasingly smaller *Ctgf* enhancer fragments, these findings suggest that although the main sequence required for notochord activity corresponds to a putative Myb-like binding site, additional binding sites for minor activators that are yet to be characterized are likely spread out along the enhancer region and contribute to the intensity of notochord staining. The *C. robusta* genome contains at least four genes encoding putative TFs of the Myb/Myb-like family and one gene encoding for a putative TF containing a SAINT/Myb domain [139]. Single-cell RNA-Seq data and our unpublished results indicate that a few of these genes might be transiently expressed in the developing *C. robusta* notochord [77,140]; it is plausible that one of these TFs might be activating the *Ctgf* notochord CRM.

Remarkably, a notochord enhancer region had been identified and partially characterized for zebrafish *ctgf* [44,141]. This region contains a putative Foxa2 binding site, as well as two additional motifs (indicated as M2 and M3) that are similar to sequences found in other enhancers with similar properties [141]. A shorter version of one of these motifs, M3, is found in the 150 bp *Cr-Ctgf* notochord CRM that we have identified; however, our mutation analysis indicates that this motif is not necessary for notochord activity in *Ciona*. A *CTGF* enhancer region active in human chondrocytes has also been described [142,143]; this region contains putative binding sites for the TFs AP1, TIE, NF1, SP-1, a CArGbox, and a TGF-β response element [142,143], none of which seem to be evidently conserved in the *Cr-Ctgf* notochord CRM described here.

In the case of the lineage-independent mosaic expression, a pattern that we first observed in the case of *multidom* [31] and is similar to that of *Ctgf* and *Ptpra*, we have proposed that one of the possible mechanisms through which this transcriptional read-out is generated might be the presence of the enhancer and suppressor of variegation chromodomain proteins, which are able to bind chromatin and modify gene expression in cell-specific contexts (e.g., [144]). As an alternative, the gradated expression of other notochord transcripts might suggest the presence of morphogen(s) emanating from the posterior end of the notochord; however, the results of experiments carried out on bisected notochords of late tailbud *Ciona savignyi* embryos argue against the existence of localized patterning cues, in either the anterior or posterior half of the notochord [24].

### 3.6. Analysis of the Cr-TGF-β Notochord CRM Provides Evidence of a Bra/Tbxt-TGF-β Axis in the Ascidian Notochord

In support of our earlier findings that suggested that Ci-Bra might control the TGF-β signaling pathway through *Cr*-Xbp1 [33], this study has provided direct evidence of a regulatory axis between Ci-Bra and the TGF-β pathway. Both genes are involved in the EMT, an event that is fundamental for normal development but also promotes the dissemination of cancer cells and the establishment of metastasis [145,146]. 

Studies in carcinoma cell lines uncovered an autocrine loop between TGF-β1 and BRA/TBXT that induces EMT [146]. Among the *Ciona* orthologs of genes involved in the TGF-β interaction network in chordoma [147], *Itgb1* (KH.C1.508) [53] and *Collagen 2A1* (also known as *CiFCol1*; KH.C7.633), are expressed in the notochord; the latter is associated with a notochord enhancer directly controlled by Ci-Bra [80]. We have previously reported the notochord expression of *olfactomedin2* (gene model: KH.C7.500), a target of TGF-β localized to the endoplasmic reticulum in other organisms (e.g., [148]), *Rb1cc1* (gene model: KH.S534.2), a mediator of autophagy [149], and *fibrillin* (gene model: KH.C3.225), which regulates the bioavailability of TGF-β by sequestering it, in its latent form, in the ECM [150]; these genes were recovered, along with *Cr-TGF-β*, in a screen for transcriptional targets of *Cr*-Xbp1 [33].

### 3.7. Evolutionarily Conserved Expression of Signaling Molecules Identified in the Ciona Notochord

We sifted through the available literature in search of evidence for the evolutionary conservation of the notochord/NP expression of the signaling genes that were identified in the *Ciona* notochord through the present study. Although the information available seemed rather fragmentary, we found a few expression reports on some of the 20 genes identified here, in addition to the information that we were able to extract from the published datasets. Expression of *Rac1* had been reported in the mouse notochord [40] and in human NP, where it had been shown to be upregulated in patients with intervertebral disc degeneration (IVDD) [151]. Also, an increase in the levels of PLCG1 in its phosphorylated state has been recently associated with IVDD and NP cell senescence [152]; similar results have been reported in the case of rat NP cells subjected to mechanical stress [153]. NCDN is expressed only in juvenile human NP [154], and its role remains to be investigated. Lastly, SEMA6A and other semaphorins were included in the molecular signature outlined for the human notochord and notochordal-like cells (NCL) derived from human induced pluripotent stem cells (hiPSCs) by a recent transcriptomic study [39]. Overall, our analysis of the published transcriptomic data for human and mouse notochord and NP shows that with the exception of Arhgap45, which was not found in the available transcriptomic datasets from neither the human notochord nor NP, the expression of the signaling molecules identified in the *Ciona* notochord through this study is conserved in the notochord and NP of mice and humans.

## 4. Materials and Methods

### 4.1. Ciona Robusta Embryo Cultures and Electroporation

Adult *Ciona robusta* (previously *Ciona intestinalis* type A; [13]) were purchased from either M-REP (Carlsbad, CA, USA) or Marinus Scientific, LLC (Long Beach, CA, USA) and kept at 18–19 °C in a refrigerated aquarium with recirculating artificial seawater. Artificial seawater (ASW) was prepared by dissolving sea salt (Tropic Marin Pro Reef, cat. no. ATM10581) in deionized water to a final salinity of 32–34 parts per thousand. Embryo cultures and electroporations were carried out as described in [31,155], with some modifications. ASW filtered through a 0.22 µm filter (FASW) was supplemented with 0.5 M TAPS pH 8.2–8.4 (FASW-T), for a final concentration of 5 mM TAPS (pH 8.2–8.4). In vitro fertilization was carried out for 5 min, by gently resuspending the gametes from 3–5 adult *Ciona* in a fingerbowl containing 300 mL of FASW-T supplemented with 3 mL of 1 M Tris-HCl (pH 9.0). Prior to dechorionation, the zygotes were transferred to another fingerbowl containing clean FASW-T, using a mesh, and washed four times with FASW-T. The dechorionation solution was prepared with FASW-T and supplemented with protease from *Streptomyces griseus* (Sigma-Aldrich, St. Louis, MO, USA; cat. no. P5145, final concentration 0.04%), sodium thioglycolate (Sigma-Aldrich, St. Louis, MO, USA; cat. no. T0632, final concentration 65.73 mM), NaOH (Fisher Scientific, Waltham, MA, USA; cat. no. S318; final concentration 42 mM), and the reaction was stopped by adding 1 mL of 10 mg/mL glycine (Sigma-Aldrich, St. Louis, MO, USA; cat. no. G8790). The embryos were gently mixed in the dechorionation solution with a Pasteur pipette for 3–6 min, allowing the chorion to detach. Prior to electroporation, dechorionated zygotes were transferred to Gelatin-coated Petri dishes and washed six times in FASW-T. For electroporation, the dechorionated zygotes were transferred to a 2-mL low-adhesion tube with ~200 µL FASW-T. The zygotes/FASW-T suspension was mixed with 500 µL of an electroporation solution, consisting of 400 µL D-Mannitol (Acros Organics, Geel, Belgium; cat. no. AC125345000; final concentration 0.77 M) and 100 µL of plasmid DNA diluted in autoclaved deionized water (ddH_2_O). The zygotes/DNA/D-Mannitol suspension was transferred to cuvettes (0.4 cm electrode, Gene Pulser, Bio-Rad, Hercules, CA, USA; cat. no. 165-2088) and the electroporations were carried out using a Bio-Rad Gene Pulser (Hercules, CA, USA) with the following settings: voltage: 50 V, pulse: 16.0 ms, number of pulses: 1, pulse interval: 0.01 s.

Zygotes were electroporated with 75–100 μg of each plasmid of interest, then cultured at either 16 °C or 20–22 °C for ~18 h and ~4.5–9 h, respectively, fixed and stained with X-Gal, essentially as previously described [31], with a few modifications. The staining reactions were performed in PBST (phosphate-buffered saline containing 0.1% Tween20) at 37 °C for approximately 24 h, using a solution containing 400 ng/mL 5-bromo-4-chloro-3-indolyl-D-galactopyranoside (X-Gal), 1 mM MgCl_2_, 3 mM potassium ferrocyanide, and 3 mM potassium ferricyanide. The reactions were stopped by washing in PBST. Each transgenic experiment was replicated at least three times under the same experimental conditions on different batches of animals.

### 4.2. Whole-Mount In Situ Hybridization (WMISH) and Gene Annotations

*Ciona robusta* embryos obtained by in vitro fertilization were fixed at stages ranging from 112-cell to late tailbud; hybridization and staining were essentially carried out as previously described [8,137]. Digoxigenin-labeled gene-specific antisense RNA probes were synthesized in vitro using as templates ESTs from the *Ciona* Gene Collection release 1 [156] and/or the *Ciona* Unigene cDNA collection [157] (Table S1) as previously described [31]. The gene names used in the figures were assigned on the basis of the results of BLASTP, BLASTX, and reciprocal TBLASTN [50] searches against the NCBI database (https://www.ncbi.nlm.nih.gov/gene/ (accessed January to September 2024).

### 4.3. Plasmid Construction

Genomic regions from the loci of the genes of interest were PCR-amplified using as templates either *Ciona robusta* genomic DNA, when isolating genomic fragments, or existing plasmid DNA, when generating truncated and/or mutated regions. PCR reactions were performed using 100–300 ng of template and High Fidelity Platinum Taq DNA Polymerase (Invitrogen, Waltham, MA, USA; cat. no. 11304011), using an Eppendorf 5333 Mastercycler (Hamburg, Germany). The thermocycling protocol included an initial denaturation at 95 °C for 2 min, followed by 30 cycles of denaturation at 95 °C for 30 s, annealing temperature for 30 s, and extension at 68 °C. The protocol concluded with a final extension at 68 °C for 5 min. PCR products were purified using the NucleoSpin Gel and PCR Clean-up Kit (Macherey-Nagel, Düren, Germany; cat. no. 740609.50). After spin-column purification, the PCR-amplified regions were cloned using T4 DNA Ligase (Promega, Madison, WI, USA; cat. no. M1801) in the pFBΔSP6 plasmid, which contains the *Foxa.a* basal promoter and the *LacZ* reporter gene and is only sporadically active in a small number of mesenchymal and muscle cells [31]. The ligated plasmids were transformed into Stellar™ Competent Cells (Takara, San Jose, CA, USA; cat. no. 636763). Plasmid DNA was purified from the transformed bacteria using the NucleoSpin Plasmid Mini Kit (Macherey-Nagel, Düren, Germany; cat. no. 740588.25) and verified by Sanger sequencing (Genewiz/Azenta, South Plainfield, NJ, USA). For medium-scale plasmid preparations (2–10 µg/ µL) intended for electroporations, the NucleoBond Xtra MIDI Kit (Macherey-Nagel, Düren, Germany; cat. no. 740410.5) was used.

All plasmids containing truncations and site-directed mutations of the notochord enhancer regions were generated by PCR amplification; the sequences of the oligonucleotide primers used to generate each construct can be found in Table S2.

### 4.4. Analysis of Gene Expression Through Published Datasets from Mouse and Human Notochord and NP

Bulk RNA-seq datasets generated using mouse notochord cells at E12.5 (*n* = 4) and NP cells at post-natal day zero (P0, *n* = 4) were obtained from the Gene Expression Omnibus (GEO) database (GSE100934) [56]. The read counts in bulk RNA-seq datasets were normalized using the DESeq2 method [158], and the normalized values were plotted as measures of gene expression. Microarray data (E-MTAB-6868; [63]) obtained from notochord cells from human embryonic (7.5–8.5-week post-conception, *n* = 3) and fetal (12–14 weeks post-conception, *n* = 2) stages were downloaded from Array Express (https://www.ebi.ac.uk/biostudies/arrayexpress accessed June–August 2024) and the intensity values were extracted and normalized. The normalized gene expression values were plotted as gene expression measures.

### 4.5. Scanpy Analysis of Published scRNA-Seq Data

Published scRNA-Seq data from *Ciona robusta* primary and secondary notochord cells [77], available from the Gene Expression Omnibus (GEO) database under accession number GSE131155, were analyzed and visualized using the Scanpy (Single-cell analysis in Python https://scanpy.readthedocs.io/en/stable/ accessed on 4 June 2024).

## 5. Conclusions

The regionalization of gene expression along the developing body axes of multicellular organisms is fundamental for the specification, segmentation, compartmentalization, and differentiation of structures with distinct morphology and function. With respect to the 40-cell *Ciona* notochord, we propose that regionalization is achieved through a combination of lineage-specific, mosaic, and gradated gene expression. Together with the different temporal onsets that we observed for the members of the evolutionarily conserved pathways analyzed here, these mechanisms can fine-tune the deployment of diverse signaling pathways across the *Ciona* notochord cells, both regionally, from anterior to posterior, and temporally, within narrow windows of expression from specification to tubulogenesis.

## Data Availability

All ESTs used for probe synthesis are listed in figures and/or in Table S1. All primers used for PCR amplifications are reported in Table S2. Original individual photomicrographs are available upon request. Human datasets: the bulk RNA-seq dataset analyzed in this study [56] is publicly available from the Gene Expression Omnibus (GEO) database (GSE100934; https://www.ncbi.nlm.nih.gov/geo/ accessed on May–September 2024); published microarray data [63] are available from Array Express (E-MTAB-6868; https://www.ebi.ac.uk/biostudies/arrayexpress accessed on May–September 2024). The single-cell RNA-Seq from *Ciona* embryos [77] analyzed in this study is publicly available from the GEO database (GSE131155).

## Supplementary Materials

The following supporting information can be downloaded at: https://www.mdpi.com/article/10.3390/ijms252413631/s1.

## Abbreviations

cDNA: complementary DNA; ChIP: chromatin immunoprecipitation; CNS: central nervous system; CRM(s): *cis*-regulatory module(s); ECM: extracellular matrix; EMT: epithelial–mesenchymal transition; EST: expressed sequence tag; FC: fold change; HD: homeodomain; hiPSCs: human-induced pluripotent stem cells; hr(s): hour(s); IVDD: intervertebral disc degeneration; kb: kilobase(s), or 1000 base pairs; MCL: Markov Clustering; ms: millisecond(s); NCL: notochordal-like cells; NP: *nuclei pulposi*; PBST: phosphate-buffered saline plus Tween; PCR: polymerase chain reaction; PIP2: phosphatidylinositol 4,5-biphosphate; PPIs: protein–protein interactions; scRNA-Seq: single-cell RNA-Sequencing; s, second(s); SH: Src homology domain; TF, transcription factor; WMISH: whole-mount in situ hybridization; WPC: weeks post-conception.
