# Supplementary material for: Fine-Tuned Expression of Evolutionarily Conserved Signaling Molecules in the Ciona Notochord
Source: Int J Mol Sci. 2024 Dec 20;25(24):13631. doi: 10.3390/ijms252413631 (PMC11728170; doi:10.3390/ijms252413631)
Supplement: Supplementary file 1 [file ijms-25-13631-s001.zip › Supplementary Files compressed/Table S1 Negron-Pineiro et al-LATEST.pdf]

Table S1. *Ciona* notochord genes encoding for signaling molecules analyzed in this study

KH Gene Model	KY Gene Model	Gene Name (ANISEED)	Best hit in human genome	Complete Gene Name	Gene Ontology (GO)	EST used to generate WMISH probe	Pre-metamorphic expression (This Study)	Pre-metamorphic expression (Previous Studies)	Post-metamorphic expression (Previous Studies)	Candidate regulator(s)
KH.C9.362	KY21.Chr9.784	RAC1; RAC2; RAC3	RAC1	Ras-related C3 Botulinum Toxin Substrate 1	GTPase activity	102C17	Fig. 1	Notochord (Coisy-Quivy et al., 2003). Not detected (32-cell, 64-cell, gastrula stages; Fujiwara et al., 2002); visceral ganglion (larva; Kusakabe et al., 2002).	Endostyle, pharyngeal gill, hemocytes, stomach, esophagus, neural gland (Ogasawara et al., 2002)	Ci-Bra*, Foxa.a*
KH.C3.493	KY21.Chr3.1093	PLCG1; PLCG2; PLCH1	PLCG1	Phospholipase C Gamma 1	phosphatidylinositol phospholipase C activity	101G20	Fig. 1	n/a	n/a	Ci-Bra#, Foxa.a*
KH.C8.67	KY21.Chr8.1114	GRAP; GRAP2; GRB2	GRB2	Growth Factor Receptor Bound Protein 2	Ras guanyl-nucleotide exchange factor activity	17e_18	Fig. 1	Not detected (32-cell, 64-cell, early gastrula, mid/late gastrula; Fujiwara et al., 2002); very weak staining in the entire body (tailbud stages; Satou et al., 2001)	Not detected (Ogasawara et al., 2002)	Foxa.a*
KH.C8.410	KY21.Chr8.1101	ARHGAP29; ARHGAP45; GMIP	ARHGAP45	Rho GTPase-Activating Protein 45	signal transduction	94E_09	Fig. 1	n/a	n/a	Ci-Bra*, Foxa.a*
KH.C4.173	KY21.Chr4.508	VANGL1; VANGL2	VANGL1	Van Gogh-Like Protein 1	transmembrane protein	92J17	Fig. 1	n/a	Ubiquitous (Ogasawara et al., 2002)	Ci-Bra#, Foxa.a*
KH.S115.7	KY21.Chr12.918	ARL13A; ARL13B; ARL2	ARL13B	ADP Ribosylation Factor Like GTPase 13B	GTPase activity	83K21	Fig. 1	n/a	n/a	Foxa.a*

KH.C6.43	KY21.Chr6.435	INPP5B; OCRL; SYNJ1	INPP5B	Inositol Polyphosphate-5-Phosphatase B	inositol polyphosphate-5-phosphatase	104i10	Figs. 1,3	Not localized (fertilized egg, 8-cell; Nishikata et al., 2001); Not detected (32-cell, 64-cell, gastrula stages; Fujiwara et al., 2002)	n/a	Ci-Bra*, Foxa.a*
KH.L9.20	KY21.Chr2.1463	PTPRA; PTPRE; PTPRS aka Ci-TWIK	PTPRA	Protein Tyrosine Phosphatase Receptor Type A	protein tyrosine phosphatase activity	64M15	Figs. 1,3	Entire body, stronger in mesenchyme (initial tailbud; Satou et al., 2001); weak expression in the trunk (tailbud stages; Satou et al., 2001); trunk endoderm (larva; Kusakabe et al., 2002)	Not detected (Ogasawara et al., 2002)	Ci-Bra***#
KH.C2.561	KY21.Chr2.220	YWHAB; YWHAG; YWHAQ	YWHAG	Tyrosine 3-Monooxygenase/Tryptophan 5-Monooxygenase Activation Protein Gamma	signal transduction	68F01	Figs. 1,3	n/a	Hemocytes, endostyle, neural complex (Ogasawara et al., 2006)	Ci-Bra**#, Foxa.a*
KH.C1.329	KY21.Chr1.75	RAB10; RAB7A; RAN	RAN	Ras-related Nuclear Protein	GTPase activity	73G06	Fig. 2	Localized (fertilized egg; 8-cell stage: A4.1, a4.2, B4.1, b4.2; Nishikata et al., 2001); ubiquitous (32-cell, 64-cell, gastrula stages; Fujiwara et al., 2002); Mesenchyme (mid-tailbud; Satou et al., 2001); endoderm (larvae (Kusakabe et al., 2002).	n/a	

KH.C4.168	KY21.Chr4.733	TBC1D15; TBC1D16; TBC1D17	TBC1D16	TBC1 Domain Family Member 16	GTPase activity	59C14	Fig. 2	n/a	n/a	
KH.C9.585	KY21.Chr9.336	EVI5L; RABGAP1; RABGAP1L	RABGAP1L	Rab GTPase-Activating Protein 1-Like	GTPase activity	21p08	Fig. 2	n/a	n/a	Ci-Bra***#, Foxa.a*
KH.C2.1078	KY21.Chr2.689	NCDN	NCDN	Neurochondrin	neuron projection development	102G24	Fig. 2	Localized (fertilized eggs, 8-cell stage: A4.1, B4.1, b4.2; Nishikata et al., 2001)	n/a	
KH.C8.800	KY21.Chr8.679	ECSIT	ECSIT	Evolutionarily Conserved Signaling Intermediate In Toll Pathway	oxidoreductase activity, acting on NAD(P)H	93D21	Fig. 2	n/a	n/a	
KH.C5.572	KY21.Chr5.311	LZTS1; LZTS2; LZTS3	LZTS2	Leucine Zipper Tumor Suppressor 2	Wnt/b-catenin pathway regulator	98N21	Figs. 2,3	Unclear, weak (initial and early tailbud; Satou et al., 2001); weak staining in the trunk (mid- and late-tailbud; Satou et al., 2001); unclear (larva; Kusakabe et al., 2002)	n/a	Ci-Bra**#
KH.C14.391	KY21.Chr14.642	SEMA6A; SEMA6B; SEMA6D	SEMA6A	Semaphorin 6A	transmembrane signaling receptor activity	95L09	Fig. 2	n/a	n/a	Ci-Bra*#, Foxa.a*
KH.C4.603	KY21.Chr4.178	LRRC8A; MFHAS1; RSU1	RSU1	Ras Suppressor Protein 1	signal transduction	89F24	Fig. 2	n/a	n/a	Ci-Bra**#
KH.C7.272	KY21.Chr7.927	HPCAL1; HPCAL4; VSNL1	HPCAL4	Hippocalcin Like 4	calcium ion binding	26n13	Figs. 2,3	n/a	n/a	Foxa.a*
KH.L71.19	KY21.Chr3.1633	APBA1; APBA2	APBA1	Amyloid Beta Precursor Protein Binding Family A Member 1	amyloid-beta binding	24i20	Fig. 2	n/a	n/a	Ci-Bra**#
KH.S256.1	KY21.Chr2.26	WEE1; WEE2	WEE1	Wee1 G2 Checkpoint Kinase	protein tyrosine kinase	29e_13	Figs. 2,3	n/a	n/a	

*Kubo et al., 2010; **Reeves et al., 2017; #Reeves et al., 2021
