# Supplementary material for: Fine-Tuned Expression of Evolutionarily Conserved Signaling Molecules in the Ciona Notochord
Source: Int J Mol Sci. 2024 Dec 20;25(24):13631. doi: 10.3390/ijms252413631 (PMC11728170; doi:10.3390/ijms252413631)
Supplement: Supplementary file 1 [file ijms-25-13631-s001.zip › Supplementary Files compressed/Table S2 Negron-Pineiro et al-FORMATTED-FINAL copy.pdf]

Table S2. Oligonucleotide primers utilized for the identification and characterization of the notochord CRMs analyzed in this study.

**Construct name, size, and ATAC-Seq peak(s) associated with it**	**Forward Primer**<br>(5'→3')<br>Restriction site: XhoI (ctcgag)	**Reverse Primer**<br>**(**5'→3')<br>Restriction site: XbaI (tctaga)
Cr-Ctgf **Notochord CRM**		
**1. Identification of the notochord enhancer region**		
5' UR (1.272 kb)<br>ATAC-Seq Peak: 8861	1272bp-F<br>atcgctcgagTAGGCGACCTTTCTTCGGC	1272bp-R<br>atgctctagaGAACACGTAAAAATGTAGAACTAACGA
Int 1 (1.142 kb)<br>ATAC-Seq Peaks: 6980, 4029	1142bp-F<br>atcgctcgagCCTCTTCTGTGTTGTCCTCTCG	1142bp-R<br>atgctctagaTTCGACAGTTCGTGTGCCTC
**2. Truncations of the 1142-bp notochord CRM**		
Int 1 (1-1039) (1.039 kb)<br>ATAC-Seq Peaks: 6980, 4029	1142bp-F<br>atcgctcgagCCTCTTCTGTGTTGTCCTCTCG	1039bp-R<br>atgctctagAAACAAATCCCCACAAAGCAGA
Int 1 (104-1142) (1.038 kb)<br>ATAC-Seq Peaks: 6980, 4029	1038bp-F<br>atcgctcgagTCGTGTGGTATGGGTTCCTC	1142bp-R<br>atgctctagaTTCGACAGTTCGTGTGCCTC
Int 1 (661-916) (378bp)<br>ATAC-Seq Peak: 4029	378bp-F<br>atcgctcgagCGGTTCGTCTGCCGTTAATAG	1039bp-R<br>atgctctagAAACAAATCCCCACAAAGCAGA
Int 1 (104-692) (588bp)<br>ATAC-Seq Peak: 6980	1038bp-F<br>atcgctcgagTCGTGTGGTATGGGTTCCTC	588bp-R<br>atgctctagaTTGTAAACAGCTATTAACGGCAGAC
Int 1 (104-356) (252bp)<br>ATAC-Seq Peak: 6980	1038bp-F<br>atcgctcgagTCGTGTGGTATGGGTTCCTC	252bp-R<br>atgctctagaCGACACGACAAAGGAAAGGC
Int 1 (362-692) (362bp)<br>ATAC-Seq Peak: 6980	362bp-F<br>atcgctcgagAACGAAGCCTTTCCTTTGTCG	588bp-R<br>atgctctagaTTGTAAACAGCTATTAACGGCAGAC
Int 1 (362-516) (185bp)<br>ATAC-Seq Peak: 6980	362bp-F<br>atcgctcgagAACGAAGCCTTTCCTTTGTCG	185bp-R<br>acgttctagaTGTGGCCATTTAGGGCCTATT
Int 1 (491-692) (202bp)	202bp-F<br>acgtctcgagAATAATAGGCCCTAAATGGCCACA	588bp-R<br>atgctctagaTTGTAAACAGCTATTAACGGCAGAC

Int 1 (542-692) (150bp)	150bp-F<br>acgtctcgagACCAAGACAACCCATTTGCG	588bp-R<br>atgctctagaTTGTAAACAGCTATTAACGGCAGAC
Int 1 (491-637) (147bp)	202bp-F<br>acgtctcgagAATAATAGGCCCTAAATGGCCACA	147bp-R<br>atgctctagaACGACTCGTTGCCGGGAC
Int 1 (542-681) (139bp)	150bp-F<br>acgtctcgagACCAAGACAACCCATTTGCG	139bp-R<br>atgctctagaTATTAACGGCAGACGAACCG
Int 1 (542-674) (132bp)	150bp-F<br>acgtctcgagACCAAGACAACCCATTTGCG	132bp-R<br>atgctctagaGGCAGACGAACCGGCA
Int 1 (542-660) (119bp)	150bp-F<br>acgtctcgagACCAAGACAACCCATTTGCG	119bp-R<br>atgctctagaGCATCAGCACTCCGG
Int 1 (542-640) (95bp)	150bp-F<br>acgtctcgagACCAAGACAACCCATTTGCG	147bp-R<br>atgctctagaACGACTCGTTGCCGGGAC
Int 1 (542-613) (72bp)	150bp-F<br>acgtctcgagACCAAGACAACCCATTTGCG	72bp-R<br>atgctctagaAACGTGAAGCTGCTAATTG
**3. Mutation Analysis**		
Int 1 (542-692) (150bp)<br>Hnf1a Mt	150bp-Hnf1a-Mt-F<br>acgtctcgagACCAAGACAACCCATTTGCGTttaCA AATATAGAGGTT	588bp-R<br>atgctctagaTTGTAAACAGCTATTAACGGCAGAC
Int 1 (542-692) (150bp)<br>HD Mt	150bp-F<br>acgtctcgagACCAAGACAACCCATTTGCG	588bp-HD-Mt-R<br>atgctctagaTTGTAAACAGCTAggAACGGCAGAC
Int 1 (542-692) (150bp)<br>Myb Mt	150bp-F<br>acgtctcgagACCAAGACAACCCATTTGCG<br>91bp-Myb-Mt-F<br>GCAGCTTCACGTgcACGGTCCCGG	83bp-Myb-Mt-R<br>CCGGGACCGTgcACGTGAAGCTGC<br>588bp-R<br>atgctctagaTTGTAAACAGCTATTAACGGCAGAC
Int 1 (542-640) (95bp)<br>Myb Mt	150bp-F<br>acgtctcgagACCAAGACAACCCATTTGCG	95bp-Myb-Mt-R<br>atgctctagaACGACTCGTTGCCGGGACCGTgcACGTG AAGCTGC

Cr-TGF-β Notochord CRM		
**1. Identification of the notochord enhancer region**		
5' UR (2.246 kb)<br>ATAC-Seq Peak: 11302	2246bp-F<br>acgtctcgagGCTAAGAGTCGCGGGGCTAC	2246bp-R<br>atgctctagaCCTCCCAACTATGAAACCGAGT
Int 1 (1.548 kb)<br>ATAC-Seq Peak: 4784	1548bp-F<br>acgtctcgagAATCGATTCGAGAGAGTGTGC	1548bp-R<br>atgctctagaGGATTCGAATGCCGCTATTG
Int 3 (1.38 kb)<br>ATAC-Seq Peak: 3846	1380bp-F<br>atcgctcgagTTATCACTGCACGGACCACG	1380bp-R<br>atgctctagAAACAAATCCCCACAAAGCAGA
**2. Truncations of the 1.38-kb notochord CRM**		
Int 3 (1-665) (665bp)	1380bp-F<br>atcgctcgagTTATCACTGCACGGACCACG	665bp-R<br>acgttctagaCGTGGTCATCACCATTATCGC
Int 3 (593-1380) (787bp)	787bp-F<br>acgtctcgagCCACAGGTATTTGTTGTTTCACGA	1380bp-R<br>atgctctagaAATACTGCATATGCGCTTCGC
Int 3 (544-682) (483bp)	483bp-F<br>atcgctcgagTACTGTGTAACGTTTGGGTGC	483bp-R<br>atgctctagaAAGTAATGCCGATTGATGCGT
Int 3 (987-1380) (393bp)	393bp-F<br>atcgctcgagACTAACGAGCACCATTATTACGC	1380bp-R<br>atgctctagaAATACTGCATATGCGCTTCGC
Int 3 (984-1111) (127bp)	127bp-F<br>acgtctcgagTTACTAACGAGCACCATTATTACGC	127bp-R<br>atgctctagaCTAGAATCCCGTTTGAAGTTTGATA
**3. Mutation Analysis**		
Int 3 (984-1111) (127bp)<br>B1 Mt	127bp-F<br>acgtctcgagTTACTAACGAGCACCATTATTACGC<br>70bp-B1Mt-F<br>CACAAAGAAAtctTCACCGCGGTGCAACTT	86-B1Mt-R<br>AGTTGCACCGCGGTGAagaTTTCTTTGTGACG<br>127bp-R<br>atgctctagaCTAGAATCCCGTTTGAAGTTTGATA

Int 3 (984-1111) (127bp)<br>B2 Mt	127bp-F<br>acgtctcgagTTACTAACGAGCACCATTATTACGC<br><br>70bp-B2Mt-F<br>CACAAAGAAAGTGTCACCGCGtctCAACTTCTC<br>GAACACAATG	92-B2Mt-R<br>TCGAGAAGTTGagaCGCGGTGACACTT<br><br>127bp-R<br>atgctctagaCTAGAATCCCGTTTGAAGTTTGATA

Abbreviations: B, Brachyury binding site; CRM, *cis*-regulatory module; HD, homeodomain; Int, intron; Mt, mutant; UR, upstream region.
